# Evaluation of Nanoparticle Uptake in Co-culture Cancer Models

**DOI:** 10.1371/journal.pone.0070072

**Published:** 2013-07-26

**Authors:** Elisabete C. Costa, Vítor M. Gaspar, João G. Marques, Paula Coutinho, Ilídio J. Correia

**Affiliations:** 1 Centro de Investigação em Ciências da Saúde (CICS), Universidade da Beira Interior, Covilhã, Portugal; 2 Unidade de Investigação para o Desenvolvimento do Interior (UDI), Guarda, Portugal; Osaka University, Japan

## Abstract

Co-culture models are currently bridging the gap between classical cultures and *in vivo* animal models. Exploring this novel approach unlocks the possibility to mimic the tumor microenvironment *in vitro*, through the establishment of cancer-stroma synergistic interactions. Notably, these organotypic models offer a perfect platform for the development and pre-clinical evaluation of candidate nanocarriers loaded with anti-tumoral drugs in a high throughput screening mode, with lower costs and absence of ethical issues. However, this evaluation was until now limited to co-culture systems established with precise cell ratios, not addressing the natural cell heterogeneity commonly found in different tumors. Therefore, herein the multifunctional nanocarriers efficiency was characterized in various fibroblast-MCF-7 co-culture systems containing different cell ratios, in order to unravel key design parameters that influence nanocarrier performance and the therapeutic outcome. The successful establishment of the co-culture models was confirmed by the tissue-like distribution of the different cells in culture. Nanoparticles incubation in the various co-culture systems reveals that these nanocarriers possess targeting specificity for cancer cells, indicating their suitability for being used in this illness therapy. Additionally, by using different co-culture ratios, different nanoparticle uptake profiles were obtained. These findings are of crucial importance for the future design and optimization of new drug delivery systems, since their real targeting capacity must be addressed in heterogenous cell populations, such as those found in tumors.

## Introduction

In the last decade the emerging development of nanomedicine has encouraged its rapid application in the development of numerous strategies to treat impairing diseases, that are still incurable [1]. Currently, the tremendous technological advances accomplished in the design and production of nanoparticulated systems for cancer therapy has originated the outcome of evermore proficient and target-specific nanocarriers, that reduce the side effects associated with classical anti-cancer therapies and also increase patient survival rate [2]. Often comprised by biocompatible inorganic or organic materials, nanocarriers are also capable of enhancing the biodistribution and bioavailability of drugs, that otherwise would be poorly available at their target sites [3]. The flexible nature of nanoparticles is intimately correlated with the various materials used for their synthesis, such as metals (gold and silver), ceramics (hydroxyapatite), lipids (cholesterol and non-toxic phospholipids) and polymers (alginate, chitosan, PEG,) [4]. Among these, chitosan has been one of the most extensively used for the synthesis of a variety of nanoparticulated drug delivery systems, due to its unique properties like biocompatibility, stability, ease to be chemically modified and low immunogenicity [5]. These unique characteristics can be further tailored by functionalizing the particle surface with cell-specific molecules such as antibodies, folic acid, biotin, or aptamers [6],[7], that significantly increase nanocarrier specificity towards target cells, protecting normal cells against drug-derived toxic side effects [8].

Nevertheless, before the plethora of nanodevices currently under investigation become suitable for clinical use, they have to surpass rigorous tests set forth by regulatory agencies such as Food and Drug Administration (FDA) and European Medicines Agency (EMEA). Included in the various evaluation stages that nanocarriers have to overcome, biological safety and activity assays assume critical relevance in nanoparticle pipeline development [9], [10].

**Figure 1:**
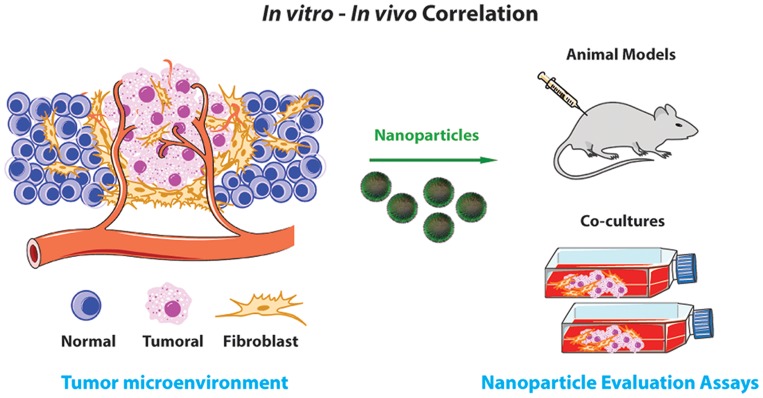
Co-culture systems are a powerful tool to test candidate drugs and delivery systems. The cell cultures are able to mimic *in vivo* tissues without the ethical and cost issues associated with animal experimentation.

Particularly, for these testing purposes, cell cultures arise as an exceptionally powerful and economic tool, in comparison to *in vivo* models. In fact, they offer a unique test platform to investigate the effects of different drug formulations and nanoparticle designs, under highly controlled and reproducible conditions [11]. Moreover, cell cultures provide an easy way to manipulate numerous experimental variables in order to reproduce some of the *in vivo* conditions, whilst avoiding ethical and legal issues associated with animal handling [12]. However, up till now the spatial organization of tissues and cell-cell interactions were commonly disregarded in the majority of the available *in vitro* models [12]. To overcome such limitations a new category of cell cultures, termed co-cultures, is currently being developed [13]. This new concept was designed with the purpose to cover the lack of correlation between classical cell cultures and *in vivo* systems (Figure 1) [12]. By using co-cultures it is possible to recreate some of the *in vivo* tissue niches [14], since cell-cell interactions are established in close contact. This critical parameter is essential for the establishment of cell morphology, phenotype, metabolism and proliferation, features that are present *in vivo* [15], [16], [17], [18]. In addition, co-cultures are also a perfect tool to analyse the targeting specificity of drug delivery systems for tumoral cells [19].

Presently, breast cancer is one of the most investigated malignancy [13]. This illness is the most common cause of cancer-related deaths in women worldwide [20], [21]. Breast cancer microenvironment is comprised not only by cancer cells but also by fibroblasts, adipocytes, blood capillaries, endothelial cells, immune system cells and extracellular matrix (ECM) proteins [13], like collagen and elastin. Despite being accepted that cancer may generally arise from genetic mutations [22], growth, invasion, and metastasis are not only dependent on these mutagenic events. Actually, it is recognized that all cells present in the tumor microenvironment act synergistically to promote tumor proliferation and spreading [23], [24]. Furthermore, it has been recently reported by Straussman et al., 2012, that stromal cells are recruited by cancer cells to prompt tumor drug-resistance and proliferation [25].

Recently, several cancer therapies that target also fibroblasts have shown to hinder this illness progression [26]. These particular cells are described as having a crucial role in the tumor niche [27], with heterogeneous populations and different relative composition existing in numerous tumors [26]. These cells are involved in the metabolism of the ECM, by promoting integrin signalling [28], [29]. Fibroblasts also secrete and interact with several growth factors [25], [29], like hepatocyte growth factor (HGF), fibroblast growth factor (FGF), insulin-like growth factor (IGF) and epithelial growth factor (EGF), that are responsible for the activation of mechanisms that contribute to apoptosis resistance [30], [31], and promote proliferation of malignant cells [32]. All these deleterious characteristics, have attracted researchers attention to develop co-cultures that include these cell types in an attempt to mimic the actual tumor microenvironment [19], [33].

From this stand point, the present study characterizes the cell uptake of functionalized nanocarriers in co-culture models, in order to address their selectivity and biological activity to cancer cells.

## Materials and Methods

### Materials

Oestrogen-dependent human breast adenocarcinoma (MCF-7) was obtained from ATCC (Middlesex, UK) and primary normal human dermal fibroblasts (hFIB) from Promocell (Heidelberg, Germany). The cell culture T-flasks were obtained from Orange Scientific (Braine-l’Alleud, Belgium). Cell imaging plates were acquired from Ibidi GmbH (Ibidi, Munich, Germany). Rhodamine B isothiocianate (RITC), Trypsin, cell culture Dulbecco’s Modified Eagle’s Medium F-12 (DMEM-F12), Collagen type I, L-histidine, L-arginine, *N*- Hydroxysuccinimide (NHS), *N*-(3-Dimethylaminopropyl)-*N*-ethylcarbodiimide hydrochloride (EDC) were purchased from Sigma–Aldrich (Sintra, Portugal). Hoescht 33342® and CellLight 2.0® BacMam were provided by Invitrogen (Carlsbad, CA, USA). Ultrapure chitosan hydrochloride (CH) (Protasan UP CL 113) was obtained from Novamatrix (Sandvika, Norway). Fetal bovine serum (FBS) was purchased from Biochrom AG (Berlin, Germany). (4-(2-hydroxyethyl)-1-piperazineethanesulfonic acid) was purchased from Tokyo Chemical Industry (TCI) (Tokyo, Japan). All other reagents were used without further purification.

### Co-culture Models

To establish different co-culture models MCF-7 and hFIB cells were grown in 75 cm^2^ cell T-flasks with DMEM-F12 medium supplemented with 10% (v/v) FBS, and 1% streptomycin and gentamycin. All cells were incubated at 37°C, in a humidified atmosphere with 5% CO_2_. Upon attaining confluence, both cells were harvested using 0.18% trypsin and 5 mM EDTA to obtain two single cell suspensions. Cells were stained with trypan blue 4% and counted by using an haemocytometer. Subsequently, co-cultures were seeded at 1∶1, 1∶3, 1∶5 and 3∶1 MCF-7 to hFIB ratios, onto 6-well plates, with a total number of 2×10^4^ of cells per well. Control homotypic cultures of hFIB and MCF-7 cells were seeded using the same total number of cells per well in separate wells. Co-cultures and homotypic cultures were maintained in DMEM-F12 complete medium throughout all experiments. The evolution of co-cultures in terms of cell distribution and morphology was analysed using an Olympus CX41 optical microscope equipped with an Olympus SP-500 UZ digital camera.

### Synthesis and Characterization of Chitosan-Histidine-Arginine

For the synthesis of the multifunctional polymer, CH was chemically modified with arginine (R) and histidine (H) through EDC/NHS coupling [34], [35]. For this purpose, chitosan was dissolved (10 mM TEMED/HCL buffer, pH 6.0) to a concentration of 1% (w/v). Subsequently, NHS, EDC (0.6 mol:1.5 mol) and L-Histidine (0.7 mol:1,0 mol glucosamine) were subsequently added to the chitosan solution. The reaction was performed during 24 h, at room temperature. The functionalized polymer was then dialyzed against deionized water (MWCO 12 000–14 000 Da). The purified chitosan-H polymer was then recovered by freeze-drying. The CH-H polymer backbone was afterwards modified with L-arginine as aforementioned, to yield CH-H-R.

The inclusion of amino acids in the chitosan backbone was analyzed through proton nuclear magnetic resonance (^1^H NMR) spectroscopy by using a Brüker Avance III 400 MHz spectrometer (Brüker Scientific Inc., N.Y., USA). All the samples were dissolved in 1 mL DMSO-d_6_ through extensive sonication and the ^1^H spectra were collected with a gradient-based pulse program (zgesgp, Brüker), at 298 K using a spectral window of 9 kHz. The NMR spectra were processed and analyzed with the TOPSPIN 3.1 software (Brüker Scientific Inc., N.Y., USA). In addition, the amino acid coupling was also verified by Attenuated Total Reflectance-Fourier Transform Infrared Spectroscopy (ATR-FTIR) (Protocol S1 in File S1 and Figure S1). The overall degree of substitution of the original CH polymer backbone was determined by ATR-FTIR, as previously described elsewere [36] (mean ± s.d.: 43.84±6.67%; *n* = 3). In addition, the degree of substitution was also determined by NMR analysis through the integration of the histidine (δ = 8.1 ppm), arginine (δ = 7.41 ppm) and chitosan (δ = 1.3 ppm) characteristic peaks (Histidine ratio = 1.14; Arginine ratio = 0.39).

### Nanoparticle Formulation and Physicochemical Characterization

To ensure the existence of genetic material for encapsulation into nanocarriers, the pVAX1-LacZ plasmid was initially amplified in recombinant bacteria and recovered as formerly reported [37]. Briefly, for the formulation of chitosan-histidine-arginine/pDNA (CH-H-R/pDNA) nanoparticles, the polymer was dissolved in acetate buffer (pH 4.5) at the desired concentration. All amino CH-H-R nanoparticles were formulated at a previously optimized amine to phosphate ratio (N:P ratio = 60). The nanoparticles were synthesized by the addition pDNA to the polymer solution at 1∶4 (v/v). The mixture was then vigorously mixed for 1 min. Afterwards, the complexes were stabilized at room temperature and recovered by centrifugation at 18 000 g, for 30 min.

Nanoparticle size and zeta potential were determined by using a Zetasizer Nano Zs instrument (Malvern Instruments, Worcestershire, UK) as our group previously described [37]. All experiments were performed at 25°C, with disposable folded capillary cells, in automatic mode. The data was processed in the Zetasizer software (v 6.2). In addition, nanoparticle zeta potential was also screened in a range of different pH’s (5.0 to 7.4), in order to further address the pH responsive behavior of this system under various conditions. The pH screening was performed by resuspending the nanoparticles in buffers with a suitable buffering capacity (Acetate buffer, 10 mM, pH 5.0; citrate buffer 10 mM, pH 6.0 and pH 6.5; HEPES buffer 10 mM, pH 7.0 and pH 7.4). All the buffers were previously filtered through a 0.22 µm filter.

The manufactured nanoparticles were also analyzed by scanning electron microscopy (SEM). Prior to SEM analysis, the nanoparticle samples were stained with 0.1% phosphotungstic acid and sonicated. Afterwards the nanoparticle suspension was dispersed in aluminum stubs, and sputter coated with gold after drying at room temperature. Nanoparticle samples were then visualized on a Hitachi S-2700 (Tokyo, Japan) electron microscope configured with optimal settings for imaging nano-sized materials.

### *In vitro* Nanoparticle Cell Uptake

The *in vitro* nanoparticle uptake in the various co-culture systems (1∶1, 1∶3, 1∶5, 3∶1) was analyzed by confocal laser scanning microscopy (CLSM). To distinguish hFIB cells from MCF-7, the latter were labelled with the CellLight 2.0® BacMam Actin-Green fluorescent protein (GFP) probe by following the manufacturer’s protocol. Prior to cell seeding, the imaging plates were surface coated with collagen I, for 30 min, as recommended by the manufacturers. After the onset of GFP expression, various MCF-7/GFP to hFIB ratios were sub-cultured on 8 well µ-Slide Ibidi plates at a density of 2×10^4^ cells per well. The cells were cultured in DMEM-F12 medium supplemented with 10% (v/v) FBS, at 37°C in humified atmosphere with 5% CO_2_. After the first day of co-culture, the cells were incubated with CH-H-R nanoparticles loaded with RITC-labelled pDNA (1 µg/cm^2^) for 4 h [38]. The nanoparticles were also incubated in monocultures of hFIB, MCF-7 and Actin-GFP MCF-7 cells. In addition, MCF-7 cells stained with GFP and incubated with naked RITC-pDNA were used as controls (Figure S2). After a 4 h incubation period, cells were fixed with 4% paraformaldehyde (PFA) for 15 min, extensively washed with PBS and stained with Hoechst 33342® nuclear probe at room temperature. Cell imaging was performed on a Zeiss LSM 710 laser scanning confocal microscope (Carl Zeiss SMT Inc., USA) by using a Plan-Apochromat 40×/1.4 Oil DIC objective. The images of the samples were acquired in z-stack mode with a slice thickness of 0.23 µm. Orthogonal sectioning and 3D reconstruction of the various z-stacks was performed in the Zeiss LSM Zen software (2010).

### Flow Cytometry Analysis of Nanoparticle Uptake

The effect of different co-culture ratios on the extent and specificity of nanoparticle uptake was analysed through flow cytometry by using a BD FACSCalibur flow cytometer (Becton Dickinson Inc., USA). Briefly, for uptake experiments, co-cultures were seeded in 6 well culture plates with a total of 2×10^5^ cells per well. Cells were grown for 24 h in DMEM-F12-10% FBS prior to all experiments. In addition, co-cultures and monocultures of hFIB and MCF-7 were used as controls to establish the correct gating and acquisition parameters in the FL-1 (530/30 nm (GFP)) and FL-2 (585/42 nm (RITC)) channels (Figure S3). For the analysis of nanoparticle uptake in the various co-cultures CH-H-R nanoparticles were prepared with freshly labeled RITC-pDNA (1 µg/cm^2^) and incubated with cells for 4 h. Afterwards, the cells were extensively rinsed with ice cold PBS and harvested with 0.18% trypsin/5 mM EDTA (Ethylenediamine tetraacetic acid). For flow cytometry analysis, the cells were resuspendend in 600 µL of fresh PBS. Data acquisition was performed in the CellQuest™ Pro software where 8×10^3^ events were recorded in the gated regions of interest assigned to hFIB and MCF-7 cells. Flow cytometry data was analyzed in the trial version of FCS express v.4 research edition software (De Novo Software, Ontario, Canada). For the calculation of cell percentage a gated region ranging from 10^1^ to 10^4^ was used. This region is delimited by a marker in the histograms and corresponds to the R2 quadrant in the dot plots. All the histograms of non-treated controls were subtracted to the histograms obtained for the different ratios.

## Results

### Co-cultures of MCF-7 and hFIB

**Figure 2:**
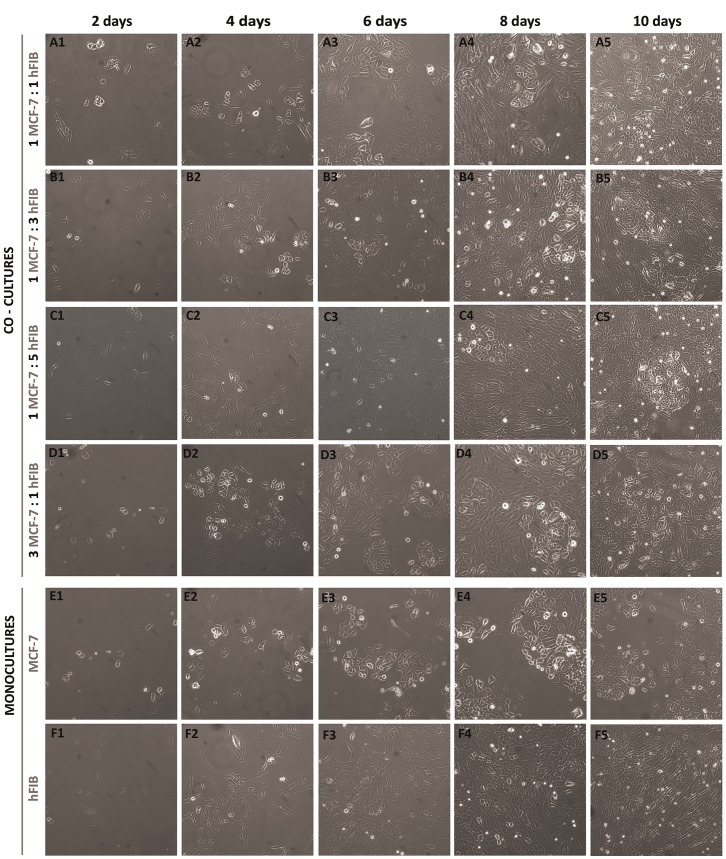
Optical images of MCF-7 and hFIB co-cultures in different ratios during 10 days of culture. Co-cultures of MCF-7 to hFIB of ratio: 1∶1 (A1-A5), 1∶3 (B1-B5), 1∶5 (C1-C5), and 3∶1 (D1-D5). Monocultures of MCF-7 (E1-E5) and hFIB (F1-F5) were used as controls. Original magnification 100×.

Initially, different breast cancer co-culture models were established using various cell ratios that were previously described in the literature as being descriptive of *in vivo*-mimicking conditions, namely, 1∶1; 1∶3; 1∶5; 3∶1, MCF-7 to hFIB cells [19], [39], [40], [41], [42]. The evolution of the established co-culture models is presented in Figure 2.

**Figure 3:**
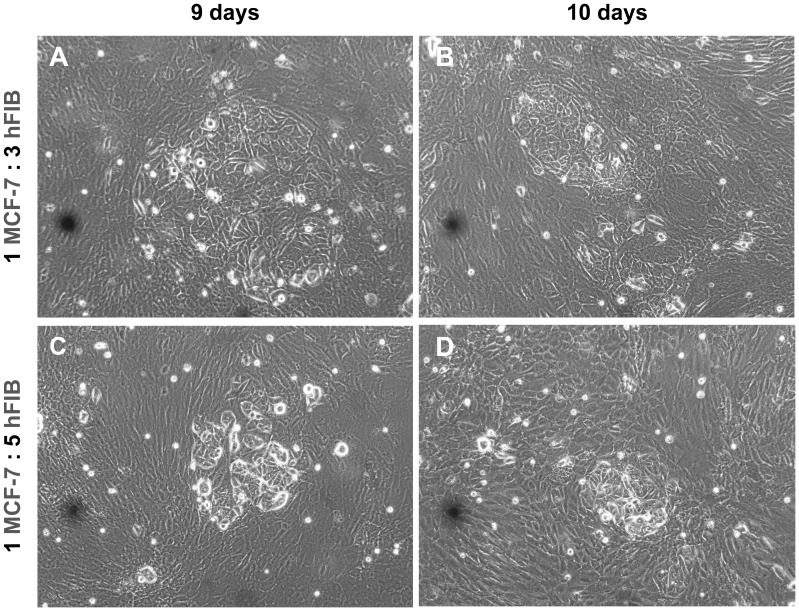
Optical images of MCF-7 and hFIB co-cultures after 9 and 10 days. Co-cultures of MCF-7 and hFIB 1∶1 (A, B) and 1∶5 (C, D). Original magnification 100×.

Through the analysis of Figure 2, it is clearly visible that cells are adherent and capable of remaining in co-culture for several days without detachment or abnormal cell death. Interestingly, in co-cultures that were established with more fibroblasts than breast cancer cells (Figure 2 B1-B5 and C1-C5), MCF-7 cells developed a slight tendency to form agglomerates surrounded by fibroblasts. This agglomeration is particularly evident after 8 days of co-culture (Figure 3) and it is associated with the phenotypic characteristics of breast cancer cells that are organized in acinar structures [43].

### Synthesis and Characterization of CH-H-R Multifunctional Polymers

**Figure 4:**
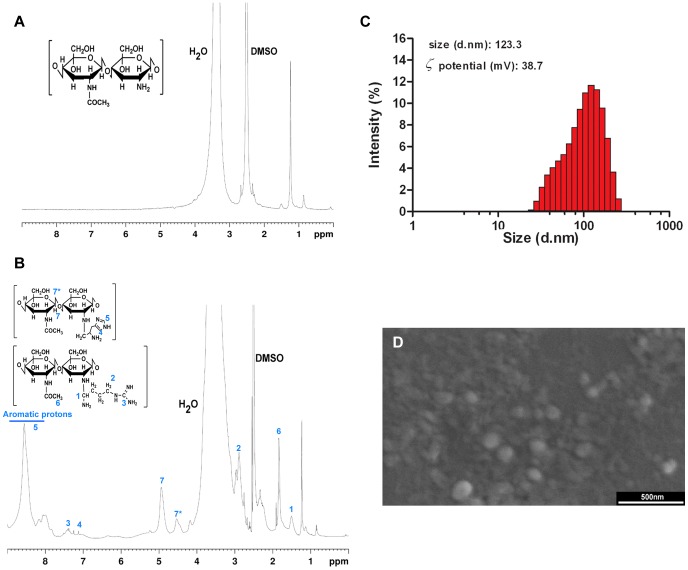
Chitosan-Arginine-Histidine and nanoparticles physicochemical characterization. ^1^H NMR spectra of unmodified CH (A) and CH-H-R (B and D). Zeta potential (C) and SEM analysis (D) of CH-H-R/pDNA nanoparticles, respectively.

The addition of amino acid residues to the chitosan backbone was confirmed by ^1^H NMR spectroscopy. The results in Figure 4 A and B demonstrate the presence of additional proton peaks at δ≈1.5 ppm (-CH_2_-, L-arginine and L-histidine, number 1) and δ≈2.8–3.0 ppm (−CH_2_NH-, L-arginine, number 2) in comparison with the spectra of chitosan alone. The signals that correspond to C_3_–C_6_ carbons of chitosan are masked by solvent peaks. The chitosan anomeric carbon signals are obtained between δ≈4.6–4.9 (number 7 and 7*) [44]. The proton signal at δ≈1.9 ppm (number 6) corresponds to the –CH_3_ groups linked to the acetamido moieties of chitosan [44]. The existence of the characteristic proton peak δ≈7.41 ppm (-NHCH = NHNH_2_, number 3) assigned to the guanidine functional group [45] present in L-arginine suggests the successful inclusion of this amino acid. In addition, the appearance of characteristic proton peaks of the L-histidine imidazole ring δ≈8.0–8.5 ppm (-N = CH-NH-, number 5) also indicates its inclusion in the chitosan polymer. Histidine also presents proton peaks at δ≈7.1–7.2 ppm that correspond to the H2 hydrogen (HN-CH-C, number 4) in the imidazole ring. These results are in agreement with those obtained by ATR-FTIR analysis (Figure S2).

### Nanoparticle Formulation and Physicochemical Characterization

**Figure 5:**
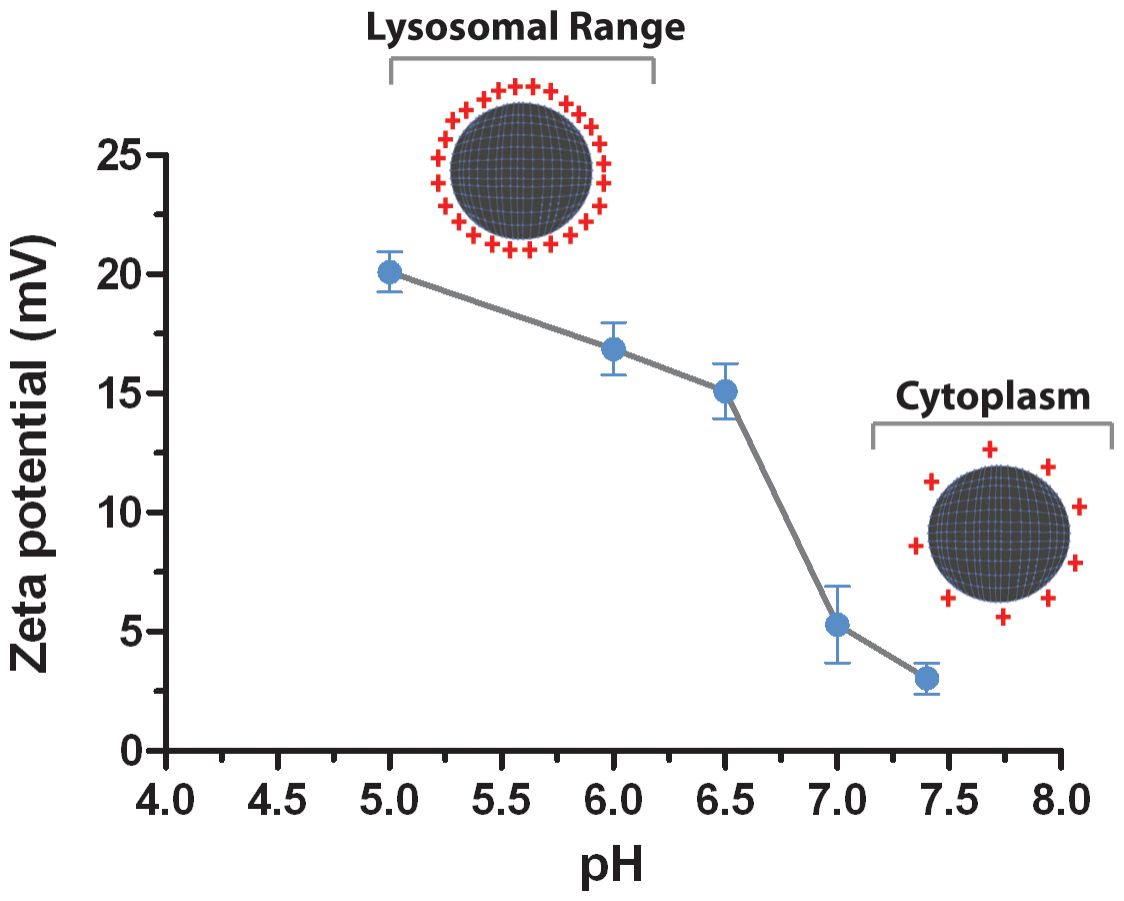
Zeta potential of CH-H-R/pDNA nanoparticles at different pH. Schematic representation of zeta potential decrease as a function of pH. Data is presented as mean ± s.d.

The synthesis of CH-H-R/pDNA nanoparticles was promoted by the establishment of attractive electrostatic forces between the positively charged polymer backbone and the negatively charged pDNA biomolecules. Nanoparticle size characterization through dynamic light scattering (DLS) revealed that the nanodevices manufactured through this process have sub-cellular size in the nanoscale range (Figure 4C), a valuable characteristic if therapeutic applications are envisioned. SEM analysis also demonstrated that particles present a spherical-like morphology (Figure 4D). Additionally, the analysis of the zeta potential revealed that the surface charge of the nanoparticles is highly positive and in the range of particle stability, *i.e.,* no particle aggregation was observed (Figure 4 C and D). The modification of the zeta potential of the nanoparticles with environmental pH was also investigated in order to further support the acquisition of a pH responsive behavior when the amino acid moieties were grafted into native chitosan (Figure 5). The obtained results demonstrate that the nanoparticles possess a high positive charge in the range of lysosomal pH (pH 5.0 to 6.0). Interestingly, at higher pH, the surface charge of the nanoparticles decreases, illustrating the deprotonation of the primary amines of chitosan and of the positively charged imidazole group of histidine (Figure 5).

### Effect of Cell Ratio on the Efficiency of Nanoparticle Cellular Uptake

**Figure 6:**
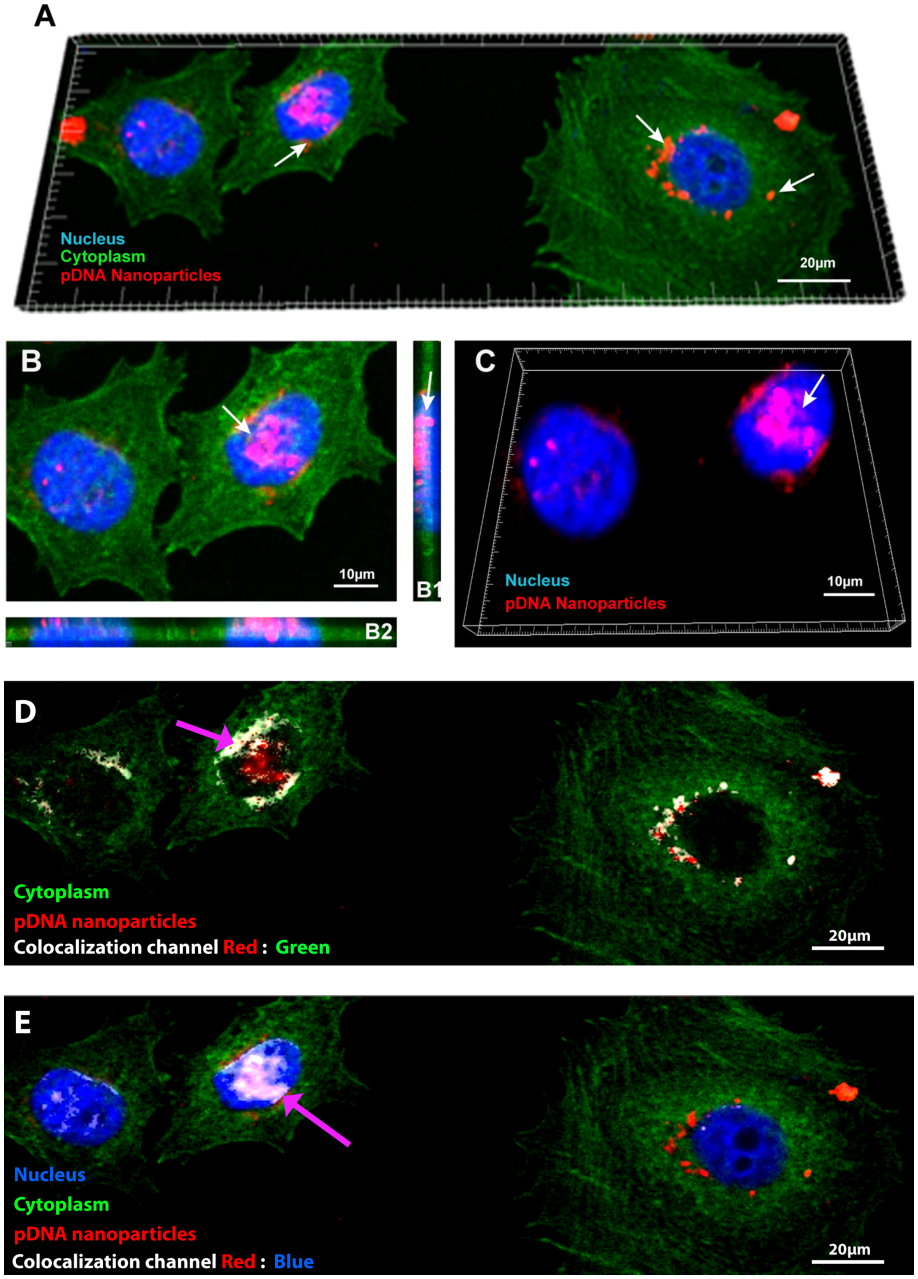
CLSM co-cultures at 1∶1 ratio for nanoparticles cellular localization analysis. Confocal microscopy images of MCF-7 breast cancer cells after 4 h of incubation with nanocarriers (A, B), orthogonal sectioning in xy axis (B1, B2), 3D reconstruction of the cell nucleus (C). Colocalization of the Red and Green channels (D). Colocalization of the Red and Blue channels. Red channel – RITC labelled pDNA/CH-H-R; Green channel – Actin-GFP staining of MCF-7 Blue Channel – Hoechst 33342® nuclear staining. Grey channels: colocalization analysis.

After confirming the successful synthesis of the nanoparticulated systems and that the MCF-7-hFIB cells remained stable in co-culture, different cell ratios were then established in co-culture with the purpose to provide a platform to evaluate cell uptake events of a multifunctional nanoparticle system comprised of CH-H-R and pDNA. Cellular uptake of the nanoparticles was initially characterized by confocal microscopy to evaluate their cell internalization capacity. The analysis of the 3D reconstruction of co-culture models demonstrate that the nanocarriers are extensively present in the cytoplasm (Figure 6 A). The pDNA-loaded nanoparticles are also localized in the cell nucleus as show in orthogonal slices (Figure 6 B, B1, B2, white arrows) and nuclear sections (Figure 6 C, white arrows). Moreover, a colocalization analysis was also performed to further corroborate these findings. As shown in Figure 6 D (colocalization channel – grey color) there is a visible colocalization between the RITC-labeled nanocarriers and the intracellular compartment (purple arrow). In addition, the nuclear colocalization also illustrates the existence of nanocarriers in the nuclear compartment.

**Figure 7:**
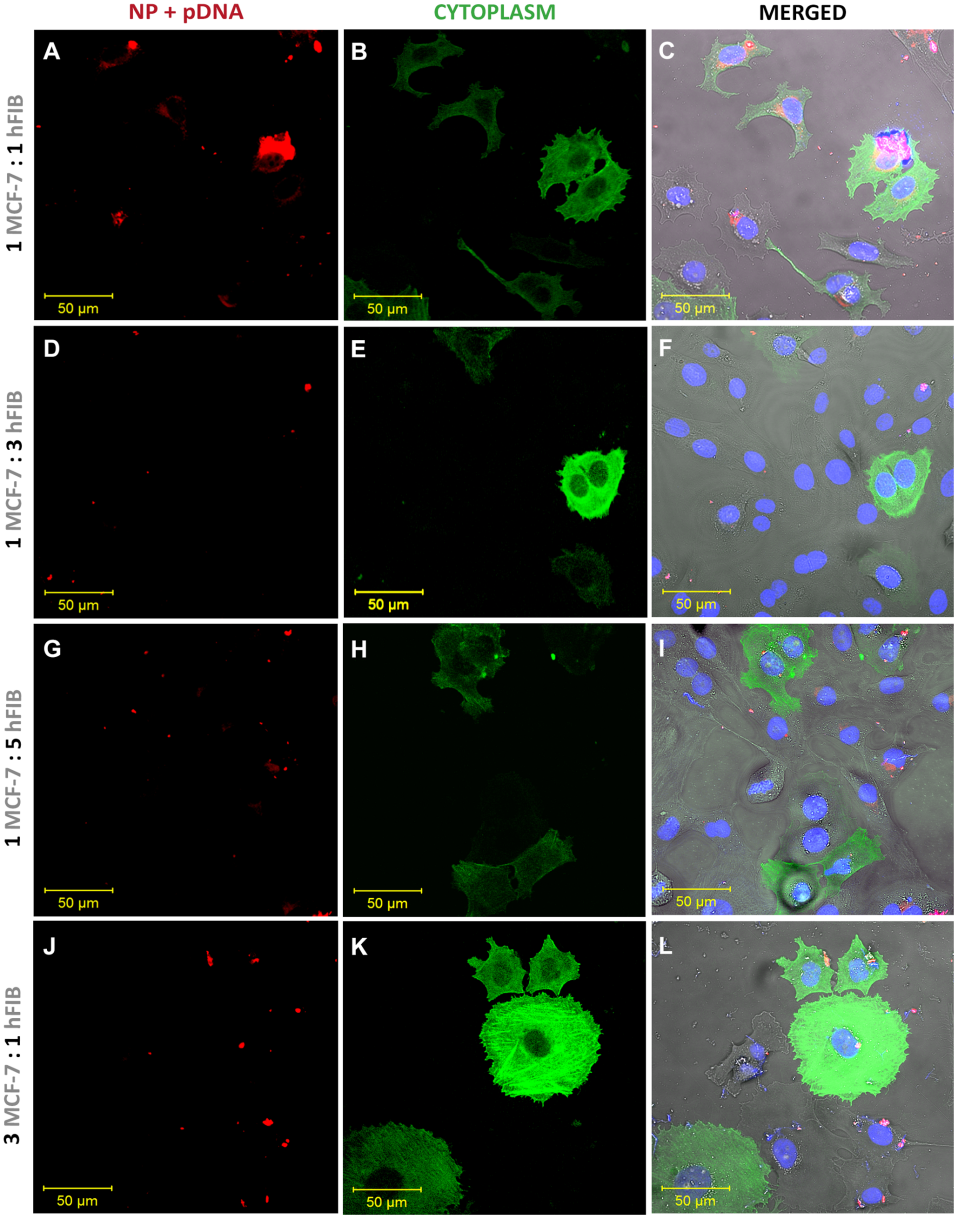
Confocal laser scanning microscopy images of nanoparticle cellular uptake co-cultures at different ratios. Co-cultures of MCF-7 to hFIB in ratios of: 1∶1 (A–C), 1∶3 (D–F), 1∶5 (G–I) and 3∶7 (J–L) after 4 h of incubation with nanoparticles. Red channel – RITC-labelled pDNA/CH-H-R nanoparticles; Green channel – Actin-GFP staining of MCF-7; Blue Channel – Hoechst 33342® nuclear staining; Grey Channel: DIC; Merged – Superimposition of all channels.

**Figure 8:**
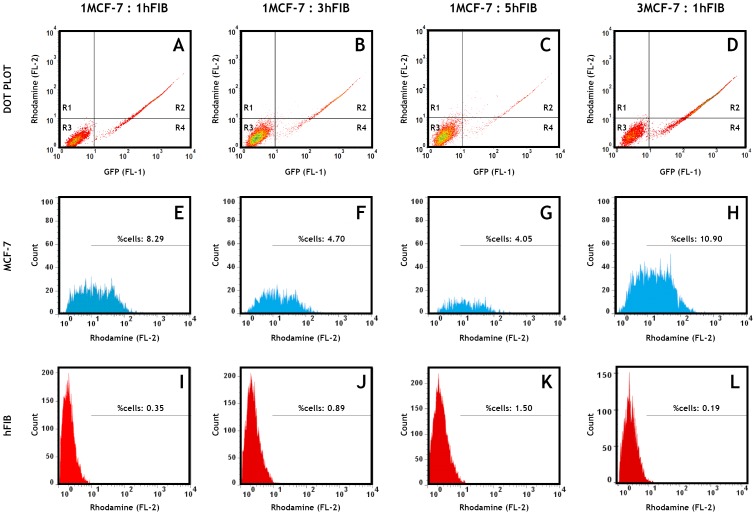
Flow cytometry analysis of nanoparticles cellular uptake in MCF-7:hFIB co-cultures. Representative dot plots of nanoparticle uptake in the different co-culture ratios (A–D). The R2 quadrant was used as a gate for histogram analysis. Representative histograms of nanoparticle uptake in MCF-7 cells (E–H) and hFIB (I–L) populations with different co-culture ratios after 4 h of incubation with RITC-labelled pDNA/CH-H-R nanoparticles.

Nanodevice targeting capacity was also evaluated in the different co-culture models by confocal microscopy and flow cytometry by labelling the pDNA present in nanoparticles with RITC and also cancer cells with a GFP-actin probe. Through this approach we were able to distinguish nanoparticle uptake in both cell types (Figure 7). Analysing the various CLSM images, we observe that for the different the co-culture ratios, the nanoparticle internalization is markedly higher in MCF-7 cells in comparison with hFIB (Figure 7). In fact, flow cytometry analysis confirms the observations of CLSM images. Figure 8 shows that the CH-R-H/pDNA nanoparticles enter preferentially in MCF-7 cancer cells, in detriment of hFIB, for all co-culture ratios. This finding emphasizes a possible selectivity of this system towards malignant cells in heterogeneous microenvironments. Moreover, nanoparticle uptake experiments in monocultured MCF-7 cells and hFIB indicate that the nanocarriers are more internalized in malignant cells than in normal cells (Figure S4), further emphasising the results obtained for the co-culture experiments.

## Discussion

Currently, the necessity to improve the efficiency of anti-cancer therapeutics has encouraged the development of proficient nanoscale delivery systems that are capable of reducing localized and systemic side effects of conventional chemotherapeutics [8]. These nanodevices possess a set of unique properties that improve the pharmacokinetic and pharmacodynamic profile of bioactive molecules, increasing their bioavailability at target sites [6]. Actually, due to their versatility, nanocarriers can be engineered to selectively recognize target tumor cells and evade first pass metabolism, phagocytosis or rapid blood clearance, decreasing the probability of off-target events [3]. In addition, once localized within the cellular compartment, nanocarriers protect their cargo from natural drug-resistance mechanisms in tumoral cells, such as those dependent on ABC transporters, and also guide the therapeutic molecules to their intracellular targets [46]. All these key characteristics are highly dependent on the different stages of nanocarrier design and assembly. Therefore, a proper evaluation during the nanoparticle development process is crucial for its successful application *in vivo.*

In fact, the evaluation of novel delivery systems must be thoroughly performed in order to prevent potential health risks and identify optimal characteristics [9]. Generally, the pre-clinical evaluation of the biological performance of a nanodevice is carried out both *in vitro*, using cell cultures, and *in vivo,* with animal models. However, the current demand to reduce animal experimentation due to the associated ethical and economic issues has prompted the development of alternative approaches such as cell cultures. However, in order to unlock the full potential of cell culture models and obtain more lifelike results, the difference between simple cell cultures and *in vivo* tissues has to be reduced. Therefore, the development of novel models based on co-cultures has brought forth the possibility to mimic tumor tissues in such a way that the physiological environment found *in vivo* can be replicated *in vitro*. Testing nanocarrier candidates in these co-cultures systems, provides more accurate results, since these reproduce tumor progression, drug resistance and also cell-cell communication [47]. All these events ultimately affect the biological performance of a given nanocarrier system and its loaded bioactive molecules.

Hence, it becomes valuable to characterize nanoparticle cellular uptake in co-culture models to evaluate their targeting specificity. Nonetheless, since the action of nanoparticles could change according to the characteristics of the tumor microenvironments, evaluating various conditions is a crucial requirement in pre-clinical development. Through this analysis the action of functionalized nanoparticles against malignant cells can then be tuned depending on the tumor niche and its surrounding normal cells. For that, it becomes essential to develop various co-culture models that reproduce dynamic environments. Thus, herein chitosan functionalized nanoparticles were tested in co-cultures with different malignant-to-normal cell ratios. To perform these assays, different ratios of hFIB and breast cancer cells (MCF-7) were used. These models were established by taking into account previous reports where these particular cell ratios were used for mimicking cancer environments [19], [39], [40], [41], [42]. Figure 2 shows the high proliferation rate of both cell types, with no abnormal cell death, compared with their monoculture counterparts. Moreover, the results shown in Figure 2 confirm the good interactions between MCF-7 breast cancer cells and hFIB, and therefore the successful establishment of these co-culture models.

Regarding cell morphologies, the optical images (Figure 2) demonstrate that both cell types preserve their phenotypic traits. Cancer cells maintained their epithelial morphology, with a polygonal shape [48], [49]. Whereas, fibroblasts show their typical spindle-shaped morphology [48], [50].

Interestingly, a dynamic change of cell structural organization with the temporal evolution of co-culture was also observed. In co-cultures with a higher number of fibroblasts than breast cancer cells, MCF-7 formed cell agglomerates surrounded by fibroblasts (Figure 3), approximately 8 days after co-culture establishment. It is important to emphasize that these aggregates present a similar organization to that of breast cancer cells obtained through biopsies [51]. Actually, MCF-7 cells reorganize themselves in structures similar to acinar architectures during co-culture [52]. These conformations are normally found in human mammary tissue [53]. In accordance with these results, previous reports also highlight the existence of distinct histological features in the course of cancer evolution [51]. The observation of this structural organization of breast cancer cells in our models further supports the reproduction of tumor-like properties *in vitro*. In 2007, Bissell, found that in addition to the presence of a cellular microenvironment, the structural organization of cells can influence their function [54]. The growth and malignant behaviour of cells appears to be regulated at the level of the overall tissue organization. Despite the fact that some co-cultures did not formed cell clusters (Figure 2 A1-A5 and E1-E5), this fact does not illustrate that these ratios of co-culture are not suitable to reproduce the tumor microenvironment. When breast cancer is in its invasive stage, cells lose their glomerular anatomy, like those presented in Figure 2 A1-A5. Indeed the majority of invasive tumours do not display characteristic features of breast cancer cells. Therefore, all these co-cultures systems, shown in Figure 2, represent viable co-culture systems that mimic different stages of breast cancer development.

Subsequently, the successful establishment of various co-cultures systems allowed the evaluation of the therapeutic capacity of the nanocarrier system tested herein. The polymeric nanoparticles used in this study are comprised by chitosan, a biocompatible polymer with positive charge that has the ability to encapsulate therapeutic nucleic acids [55]. This characteristic renders it an ideal biomaterial for gene delivery [55]. Moreover, to increase the biological activity and selectivity of the system, the polymeric backbone of chitosan was also conjugated with two functional and bioinspired ligands, namely, arginine and histidine. The conjugation of chitosan with these ligands to yield the multifunctional CH-H-R polymer was demonstrated by proton H^1^ RMN spectroscopy through the existence of the characteristic proton peaks assigned to the functional groups of both amino acids.

Following polymer synthesis, the physicochemical characterization of the pDNA-loaded nanoparticles revealed that these delivery systems have sizes suitable for tumor accumulation and cell uptake. Moreover, the overall positive zeta potential additionally contributes to the capacity of nanoparticles to interact with target cells. These suitable physicochemical characteristics and additional features are attributed to the conjugation of chitosan with histidine and arginine.

Arginine has been recently described as a valuable ligand, since it increases nanoparticle uptake through the establishment of electrostatic interactions with the negatively charged cancer cell membranes [56]. In addition, the chitosan-based delivery system tested is also composed of histidine moieties that improve the endosomal release capacity of this nanocarrier. Endosomal release has been described as one of the most rate limiting stages in the delivery of bioactive molecules encapsulated in nanoparticles, since they can become trapped inside these vesicles, a fact that significantly decreases their therapeutic efficacy [57]. The presence of histidine residues presents additional advantages in the acidic microenvironment that surrounds tumors since this amino acid has a pH-responsive behaviour becoming positively charged at mildly acidic conditions, increasing therefore the cationic surface charge of the nanodevices, in fact this valuable behaviour is corroborated by the zeta potential experiments performed at different pHs, which demonstrate that in the lysosomal pH range the nanocarriers are highly positively charged (Figure 5) [57]. Histidine also promotes an additional pH-sensitive shift in the cell cytoplasm, becoming neutral at physiological pH, this fact in conjugation with the deprotonation of the chitosan primary amines also at physiological pH thus contributes to a lower zeta potential, as demonstrated in Figure 5. This interesting responsive profile is ultimately responsible for the possible onset of biomolecule release in the intracellular compartment.

These important features improve the cellular uptake of this delivery system in different co-culture models. Actually, as shown by CLSM images the nanoparticles are extensively localized in breast cancer cells nucleus (Figure 6). Additionally, CLSM images show that nanoparticle uptake is noticeably higher in MCF-7 tumor cells than in hFIB, for all ratios tested (Figure 7). These results are in agreement with those obtained by flow cytometry-based population analysis that demonstrates a higher percentage of cancer cells with nanoparticles than hFIB in all co-culture models (Figure 8). In other hand, confocal images (Figure S2 A–C and D–F) and flow cytomtry results (Figure S4) of nanoparticles cell uptake in monocultures, also demonstrate an higher penetration of the chitosan nanodevices in MCF-7 than hFIB. These results suggested that this particular nanodevice possesses valuable characteristics when administered in the complex tumor microenvironment, since it delivers its cargo with more efficiency to malignant cells, thus contributing for an improved therapeutic outcome. This capacity is most likely correlated with the amino acid functionalization that allows these systems to interact more with negatively charged proteoglycans commonly over-expressed in cancer cells [58].

Another important event demonstrated by flow cytometry is the influence of the number of hFIB in co-culture in the extent of nanoparticle uptake. Namely, with the increasing number of normal cells (hFIB) in the ratios 1∶3 and 1∶5 less cancer cells internalized nanoparticles (Figure 8 J and K), in comparison with the 1∶1 ratio (Figure 8 I). Whereas hFIB cells had internalized a slightly higher number of particles in comparison with the 1∶1 ratio (Figure 8). Reciprocally, in the 3∶1 ratio the presence of more malignant cells led to an increased MCF-7 nanoparticle uptake (Figure 8 L), with hFIB having internalized far less nanocarriers (Figure 8 H).

These important differences in normal and cancer cells nanoparticle uptake draw attention to the need of using various models to properly mimic tumor heterogeneity and investigate the biological efficiency and specificity of a nanocarrier system in different tumor environments. In fact, the recent reports that employ co-culture models to evaluate the biological efficiency of nanodevices, disregard the possible uncertainty of the cell populations present in a tumor microenvironment [19], thus, not exploring the full potential of this testing platform. Our results demonstrate that the same nanoparticle formulation could transfect different percentage of cancer cells and normal cells, depending on their co-culture ratios.

This is important to improve nanoparticle selectivity in heterogeneous tumors and emphasizes the necessity of evaluating nanoparticle performance in different co-culture models to mimic, as close as possible, the tumor scenarios that might be found in different patients. Particularly, for the system tested herein the results obtained also revealed that the functionalized nanoparticles are capable of escaping endosomal vesicles and enter into the nuclear compartment. Such allows us to postulate that these nanocarriers could be applied in the delivery of bioactive therapeutics with anti-tumoral activity. In fact, these systems are loaded with pDNA biomolecules that could in the future encode pro-apoptotic agents or tumor suppressor genes, and thus elicit a therapeutic effect.

### Conclusion

The results obtained in this work, showed that it is possible to mimic breast cancer microenvironments with the establishment of different cell co-culture models. The MCF-7 cells grew in the presence hFIB for the different ratios used herein. Acinar-like cell clusters were observed for some of the co-cultures, which reproduce the microenvironment found in breast cancers. Actually, these agglomerates are characteristic of histologic sections obtained in biopsies of breast cancer tissues. It is also important to emphasize that, co-cultures represent descriptive, simple, and inexpensive systems to evaluate the biological performance of nanoparticles in microenvironments that closely mimic the tumor niche. Addressing this heterogeneity is crucial since different malignant-to-normal cell ratios have influenced nanoparticle uptake.

In conclusion, the obtained results provide an important foundation for future studies that aim to evaluate the biological activity of a new drug or gene delivery system in experimental conditions, close to those found inside the human body. The nanocarriers evaluated in these co-culture systems will be used, in a near future, to encapsulate pDNA encoding genes for cancer therapy. Moreover, in future works, all these co-culture models may also be established in three dimensional matrices, in order to obtain organotypic structures.

## Supporting Information

Figure S1 **ATR-FTIR spectra of CH and CH-H-R polymers.** Blue spectra: CH polymer. Red spectra: CH-H-R modified polymer. The modification of the native polymer by the inclusion of amino acids through a selective amidation process is confirmed by the increased peak intensity in the amide I band (1630–1665 cm^−1^) of the CH-H-R spectra.
(TIF)

Figure S2 **CLSM images of MCF-7 and hFIB monocultures incubated with CH-H-R/pDNA nanoparticles and free pDNA.** Cultures of hFIB (A–C), MCF-7 (D–F) and MCF-7 non-stained (G–I) after incubation with nanoparticles. Red channel – Rhodamine B labeled pDNA/CH-H-R nanoparticles. Green channel – Actin-GFP MCF-7 cells; Blue Channel – Cell Nucleus (Hoechst 33342®); Grey Channel – Differential interference contrast (DIC); Merged – Superimposition of all channels. Both MCF-7 (Actin-GFP and non-stained) and hFIB mono-cultured cells internalize nanoparticles. RITC-pDNA alone is unable to transpose the extracellular barriers (J–L).
(TIF)

Figure S3 **Controls of flow cytometry analysis of mono and co-cultures non incubated with nanoparticles.** Representative histograms of non-stained and non-incubated MCF-7 and hFIB monocultures: FL-1 GFP channel (A, B), and FL2-Rhodamine channel (C and D), respectively; Representative histograms of non-stained and non-incubated MCF-7 and hFIB co-cultures at various ratios: FL-1 GFP (E, F, G and H) and FL-2 Rhodamine (I, J, K and L).
(TIF)

Figure S4 **Nanoparticle uptake in monocultures by flow cytometry.** Monoculture of MCF-7 cells (A). Monocultures of hFIB (B). Marker line represents the gated region used for data analysis.
(TIF)

File S1 (DOC)
