# Supplementary figures and images for: Evaluation of Nanoparticle Uptake in Co-culture Cancer Models
Source: PLoS One. 2013 Jul 26;8(7):e70072. doi: 10.1371/journal.pone.0070072 (PMC3724604; doi:10.1371/journal.pone.0070072)

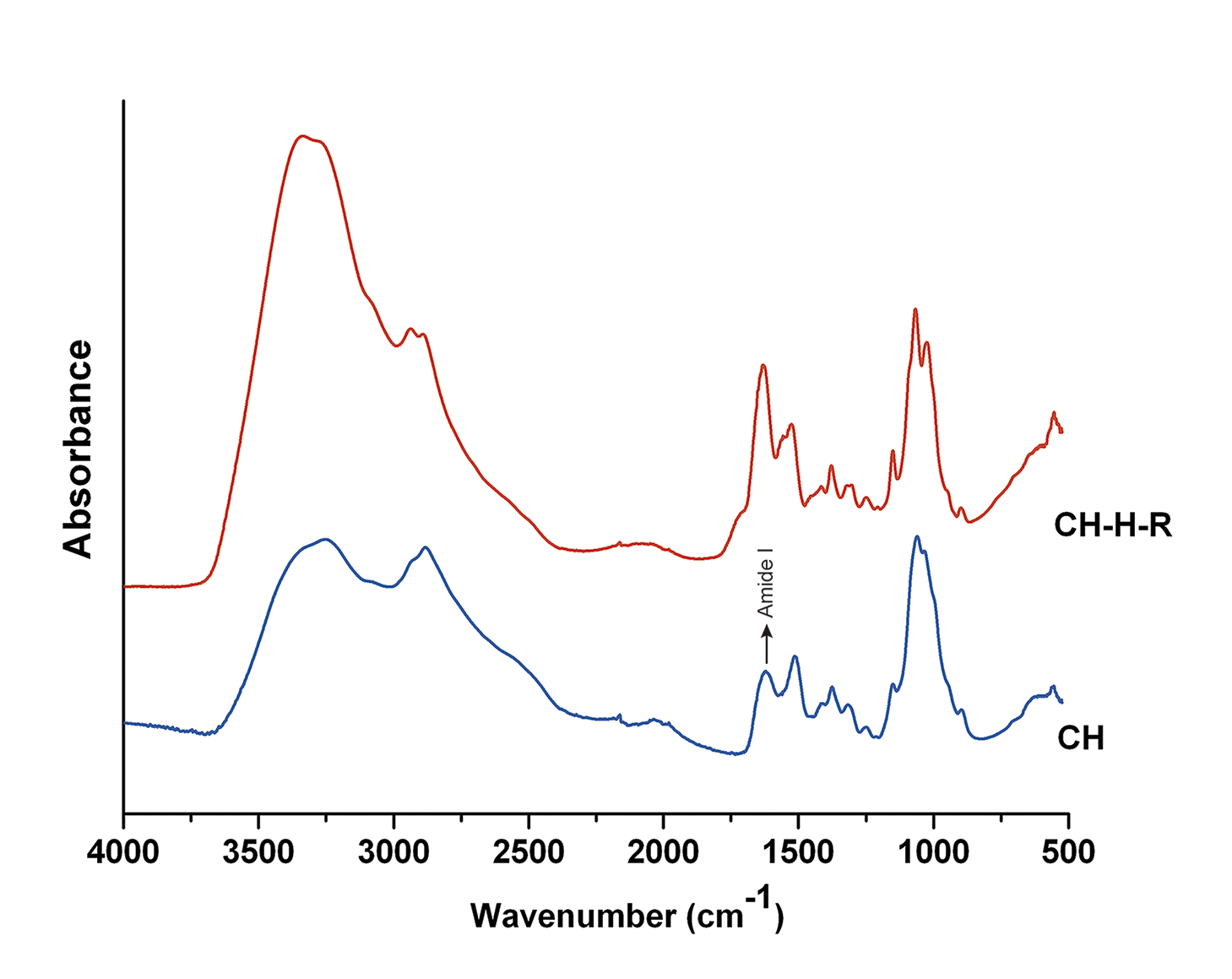

Supplement: Figure S1 — ATR-FTIR spectra of CH and CH-H-R polymers. Blue spectra: CH polymer. Red spectra: CH-H-R modified polymer. The modification of the native polymer by the inclusion of amino acids through a selective amidation process is confirmed by the increased peak intensity in the amide I band (1630–1665 cm−1) of the CH-H-R spectra. (TIF) [file pone.0070072.s001.tif]

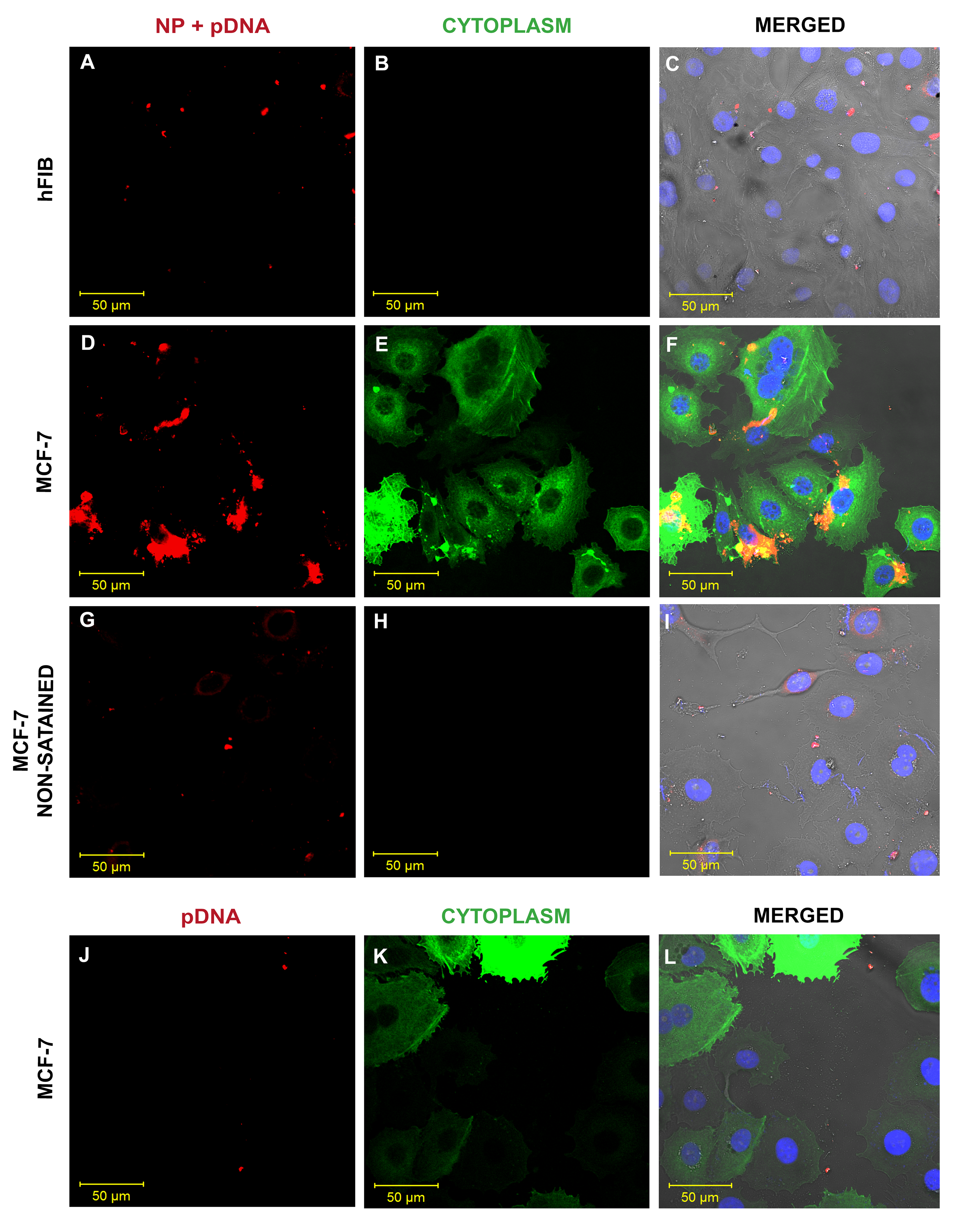

Supplement: Figure S2 — CLSM images of MCF-7 and hFIB monocultures incubated with CH-H-R/pDNA nanoparticles and free pDNA. Cultures of hFIB (A–C), MCF-7 (D–F) and MCF-7 non-stained (G–I) after incubation with nanoparticles. Red channel – Rhodamine B labeled pDNA/CH-H-R nanoparticles. Green channel – Actin-GFP MCF-7 cells; Blue Channel – Cell Nucleus (Hoechst 33342®); Grey Channel – Differential interference contrast (DIC); Merged – Superimposition of all channels. Both MCF-7 (Actin-GFP and non-stained) and hFIB mono-cultured cells internalize nanoparticles. RITC-pDNA alone is unable to transpose the extracellular barriers (J–L). (TIF) [file pone.0070072.s002.tif]
