# Supplementary material for: Evaluation of Nanoparticle Uptake in Co-culture Cancer Models
Source: PLoS One. 2013 Jul 26;8(7):e70072. doi: 10.1371/journal.pone.0070072 (PMC3724604; doi:10.1371/journal.pone.0070072)
Supplement: File S1 — (DOC) [file pone.0070072.s005.doc]

**Evaluation of Nanoparticle Uptake in Co-culture Cancer Models**

**Elisabete C. Costa^1^, Vítor M. Gaspar^1^, João G. Marques^1^, Paula Coutinho^1,2^, Ilídio J. Correia^1^***

**1** Centro de Investigação em Ciências da Saúde (CICS), Universidade da Beira Interior, 6200-506, Covilhã, Portugal, **2** Unidade de Investigação para o Desenvolvimento do Interior (UDI), 50 6300-654, Guarda, Portugal

*Corresponding-Author: Prof. Ilídio J. Correia.

CICS-UBI - Centro de Investigação em Ciências da Saúde, Faculdade Ciências da Saúde, Universidade da Beira Interior, Avenida Infante D. Henrique, 6200-506, Covilhã, Portugal

E-mail: [icorreia@ubi.pt](mailto:icorreia@ubi.pt)

**SUPPLEMENTARY INFORMATION**

**Protocol S1**

**Attenuated Total Reflectance - Fourier Transform Infrared Spectroscopy (ATR-FTIR)**

The initial characterization of the polymer modification with both amino acids was investigated by ATR-FTIR spectroscopy. Prior to all experiments the polymers were freeze dried and stored in a vacuum oven until analysis. The infrared data was collected in a Nicolet iS10 IR spectrometer (Thermo Scientific Inc., MA, USA) at room temperature with the diamond-ATR correction mode. The interferograms were recorded with a 4 cm^-1^ spectral resolution and a spectral width of 500-4000 cm^-1^ in high resolution mode with atmospheric suppression. For each experiment at least 256 interferograms were acquired.
